# Systematic review of terminology, definitions, and eligibility criteria in trials of neonatal encephalopathy, hypoxic-ischemic encephalopathy, and perinatal asphyxia

**DOI:** 10.1038/s41390-025-04750-2

**Published:** 2026-02-27

**Authors:** Tim Hurley, Fiona Quirke, Aoife Branagan, Robert McCarthy, Elaine Finucane, Graham King, Petek Eylul Taneri, Mohamed El-Dib, Frank Harry Bloomfield, Beccy Maeso, Betsy Pilon, Sonia Bonifacio, Courtney J. Wusthoff, Lina Chalak, Cynthia F. Bearer, Deirdre M. Murray, Nadia Badawi, Suzann K. Campbell, Sarah B. Mulkey, Pierre Gressens, Donna M. Ferriero, Linda S. de Vries, Karen Walker, Sarah Kay, Geraldine B. Boylan, Christopher Gale, Nicola J. Robertson, Mary D’Alton, Tetyana H. Nesterenko, Ulrike Mietzsch, Alistair J. Gunn, Karin B. Nelson, Declan Devane, Eleanor J. Molloy, Steering Group for DEFiNE (Definition of Neonatal Encephalopathy), AAoife Branagan, Tim Hurley, Fiona Quirke, Declan Devane, Petek E. Taneri, Nadia Badawi, Cynthia F. Bearer, Frank H. Bloomfield, Sonia L. Bonifacio, Geraldine Boylan, Suzann K. Campbell, Lina Chalak, Mary D’Alton, Linda S. de Vries, Mohamed El-Dib, Donna M. Ferriero, Chris Gale, Pierre Gressens, Alistair J. Gunn, Sarah Kay, Deirdre M. Murray, Karin B. Nelson, Betsy Pilon, Nicola J. Robertson, Karen Walker, Courtney J. Wusthoff, Eleanor J. Molloy

**Affiliations:** 1 Department of Pediatrics, School of Medicine, Trinity College Dublin, Dublin, Ireland; 2 Trinity Translational Medicine Institute, Trinity College Dublin, Dublin, Ireland; 3 Trinity Research in Childhood Centre (TRiCC), Centre, Ireland; 4 Health Research Board Neonatal Encephalopathy PhD Training Network (NEPTuNE), Dublin, Ireland; 5 Health Research Board – Trials Methodology Research Network (HRB-TMRN), University of Galway, Galway, Ireland; 6 School of Nursing and Midwifery, University of Galway, Galway, Ireland; 7 School of Medicine, University of Limerick, Limerick, Ireland; 8 Department of Paediatrics, The Coombe Hospital, Dublin, Ireland; 9 Evidence Synthesis Ireland, University of Galway, Galway, Ireland; 10 Trinity College Institute of Neuroscience, Trinity College Dublin, Dublin, Ireland; 11 Cochrane Ireland, University of Galway, Galway, Ireland; 12 Department of Pediatric Newborn Medicine, Brigham and Women’s Hospital, Harvard Medical School, Boston, MA USA; 13 Liggins Institute, University of Auckland, Auckland, New Zealand; 14 James Lind Alliance, School of Healthcare Enterprise and Innovation, University of Southampton, Southampton, UK; 15 Hope for HIE, West Bloomfield, MI USA; 16 Division of Neonatal and Developmental Medicine, Department of Pediatrics, Stanford University School of Medicine, Palo Alto, CA USA; 17 Department of Neurology, University of California- Davis, Sacramento, CA USA; 18 Division of Neonatal-Perinatal Medicine, University of Texas Southwestern Medical Center, Dallas, TX USA; 19 Division of Neonatology, Department of Pediatrics, Rainbow Babies & Children’s Hospital, Cleveland, OH USA; 20 Case Western Reserve University School of Medicine, Cleveland, OH USA; 21 INFANT Research Centre, Cork, Ireland; Department of Pediatrics and Child Health, University College Cork, Cork, Ireland; 22 Cerebral Palsy Alliance Research Institute, Specialty of Child & Adolescent Health, Sydney Medical School, Faculty of Medicine & Health, The University of Sydney, Sydney, NSW Australia; 23 Grace Centre for Newborn Intensive Care, Sydney Children’s Hospital Network, The University of Sydney, Westmead, NSW Australia; 24 Department of Physical Therapy, College of Applied Health Sciences, University of Illinois Chicago, Chicago, IL USA; 25 Prenatal Pediatrics Institute, Children’s National Hospital, Washington DC, VA USA; 26 Department of Neurology, The George Washington University School of Medicine and Health Sciences, Washington DC, VA USA; 27 Department of Pediatrics, The George Washington University School of Medicine and Health Sciences, Washington DC, VA USA; 28 Université Paris Cité, NeuroDiderot, Inserm, Paris, France; 29 Department of Pediatrics and Neurology, University of California San Francisco, Weill Institute for Neurosciences, San Francisco, CA USA; 30 Department of Neonatology, Wilhelmina Children’s Hospital, University Medical Center Utrecht, Utrecht, The Netherlands; 31 Department of Newborn Care, Royal Prince Alfred Hospital, Sydney, Local Health District, Sydney, NSW Australia; 32 Faculty of Medicine and Health, University of Sydney, Sydney, NSW Australia; 33 PEEPS-HIE, Manchester, UK; 34 Neonatal Medicine, School of Public Health, Faculty of Medicine, Chelsea and Westminster Campus, Centre for Paediatrics and Child Health, Imperial College London, London, UK; 35 Institute for Women’s Health, University College London, London, UK; 36 Centre for Clinical Brain Sciences, University of Edinburgh, Edinburgh, UK; 37 Department of Obstetrics and Gynecology, Columbia University, New York, NY USA; 38 Department of Neonatology, Cleveland Clinic Children’s Hospital, Cleveland, OH USA; 39 Division of Neonatology, Department of Pediatrics, University of Washington Medical School, Seattle Children’s Hospital, Seattle, WA USA; 40 Departments of Physiology and Paediatrics, School of Medical Sciences, University of Auckland, Auckland, New Zealand; 41 National Institutes of Health, National Institute of Neurological Diseases and Stroke, Bethesda, MD USA; 42 Department of Neonatology, Children’s Hospital Ireland at Crumlin and Tallaght, Dublin, Ireland

## Abstract

**Background:**

Appropriate terminology and definitions of neonatal encephalopathy (NE), hypoxic-ischemic encephalopathy (HIE), and perinatal asphyxia (PA) remain controversial. Participant criteria used in therapeutic hypothermia (TH) trials are frequently used as case definitions for NE/HIE/PA but studies are inconsistent. This review aims to assess variations in terminology and case participant criteria between trials for NE/HIE/PA.

**Methods:**

Search strategy retrieved articles from databases (Embase, MEDLINE, CENTRAL, CDSR and WHO) for randomized controlled trials (RCTs) of interventions for NE/HIE/PA using any definition for NE/HIE/PA. Outcomes were a description of the terminology, definitions, and participant criteria. Two reviewers independently screened results. Qualitative results were synthesized in a narrative summary.

**Results:**

The search provided 6768 results. 67 were included in the qualitative synthesis. HIE was the most frequently used term (56/67). NE was the least frequent (16/67). Some of the common inclusion criteria were Apgar scores (63/67), metabolic acidosis (58/67), and reduced level of consciousness (57/67). Most frequently employed exclusion criteria were prematurity (63/67), major congenital abnormalities (62/67), and identification beyond 6 h from birth (62/67).

**Discussion:**

This review identified variations in terminology and in-trial participant criteria between studies. These results will inform a consensus process for developing a definition and case definition of NE/HIE/PA.

**Impact:**

- Our article demonstrated significant variations in the terminology used to describe the condition of NE/HIE/PA, which demonstrates a need for more consistent definitions in terminology.
- A broad but meaningful definition of the condition would provide an inclusive approach while permitting subclassifications within the condition, and permitting comparisons and benchmarking across different settings.
- Developing consistency across these areas, as far as possible, would allow improved interpretation of interventions on long-term prognosis and greater generalizability of trial results

## Introduction

Neonatal encephalopathy (NE)/hypoxic ischemic encephalopathy (HIE)/perinatal asphyxia (PA) are generally understood to be associated with disturbed neurological function in term or late preterm neonates in the first few days of life.^1^ For some, HIE is a distinct entity and has been defined as a cause-specific subgroup of NE caused by inadequate blood flow and oxygen delivery to the brain.^2^ Many, including the American Academy of Pediatrics, have advocated for NE to be used, as it does not assume an etiology, and HIE to be limited to a cause-specific subset; however, this is not universally accepted. Despite this distinction, many use the term HIE in preference to NE to refer to the broader condition or use the terms interchangeably. PA often refers to impaired gas exchange in the intrapartum or immediate postnatal period,^3^ although some use the term to refer to the clinical entity of NE/HIE/PA. There is no specific diagnostic test for NE/HIE/PA, and it is diagnosed based on generally accepted clinical and biochemical findings. The condition has a multifactorial etiology^4^ and is associated with multi-organ dysfunction.^5^ Some argue that NE is an inadequate term for the condition,^1^ and appropriate terminology and diagnostic criteria for the condition have been an ongoing debate for over 20 years with no conclusion or consensus reached.^6–10^

Although many infants are exposed to PA, only a proportion of these will have neurological dysfunction, and some will not have significant early signs of PA but will have significant neurological dysfunction.^11^ Identifying infants eligible for therapeutic hypothermia (TH) within the first six hours from birth is essential and is the gold standard treatment in high-resource settings.^12^

In the absence of consensus terminology or diagnostic criteria for NE/HIE/PA, the case definition has been implied from the eligibility criteria for trials of NE/HIE/PA.^13,14^ While this provides clarity, it also may drive a restrictive definition that does not include all patients with the condition nor all those at risk of adverse outcomes. For example, RCTs of TH were limited to those with moderate to severe NE/HIE/PA and trial eligibility criteria reflected this. However, there is a spectrum of severity of NE/HIE/PA, and this definition excludes those with mild NE/HIE/PA, although it is evident that they are at increased risk of adverse outcomes compared to the general population.^15,16^ There is controversy about the use of TH for patients with mild NE/HIE/PA^17^ despite the lack of evidence to date for benefit.^18^ Thus, although patients with mild NE/HIE/PA may not be eligible for TH, they are at increased risk of adverse outcomes and therefore should be included in any definition of the condition and should be considered for future trials of therapies for NE/HIE/PA. Furthermore, a direct comparison of the application of the eligibility criteria from the National Institute of Child Health and Human Development Neonatal Research Network (NICHD-NRN) and Total Body Hypothermia-British Association of Perinatal Medicine (TOBY-BAPM) trials demonstrated that subtle differences in standardized examinations used to define NE/HIE/PA resulted in a significant difference in the number of infants eligible for TH.^19^ There is a need for standardization of the criteria for TH and the neurological exam in particular,^20^ and more broadly, for the inclusion of those diagnosed with mild NE/HIE/PA.

This systematic review describes the frequency of terminology, definitions, and the eligibility criteria used for NE/HIE/PA in clinical trials. It identifies similarities and differences between trials, describes how this may impact control group mortality between trials, and ultimately provides the background to develop consensus terminology, definition, and eligibility criteria for trials of interventions for patients with NE/HIE/PA.

## Methods

This study was developed as an extension of a registered protocol with Prospero (CRD42020170265), a systematic review of reported outcomes in RCTs in NE/HIE/PA. This systematic review was conducted in accordance with Preferred Reporting Items for Systematic Reviews and Meta-analyses (PRISMA) reporting guideline^21^ and the published protocol apart from search and screening methodologies. The manuscript was initially drafted (TH, EM) and disseminated to the wider author group. Iterative amendments to the draft were included, however the analysis and results review were conducted as stated in the methodology.

### Information sources and search strategy

We systematically searched five major databases: Embase; MEDLINE; Cochrane Central Register of Controlled Trials (CENTRAL); Cochrane Database of Systematic Reviews (CDSR), and the World Health Organization (WHO) in April 2020, updated in November 2022, to identify all relevant trials. Additional data sources were identified through screening all relevant primary studies and review articles and a discussion with experts in this area. Only papers available in English were included. We used MESH and search terms to identify RCTs in NE, hypoxic-ischemic encephalopathy, or PA. The completed search strategy and full search terms are available in Supplementary Methods S1. Authors were contacted for additional details not included in the publication if they were required for inclusion.

### Eligibility criteria

Population: Infants diagnosed with NE/HIE/PA within the neonatal period. Studies that included participants <34 weeks’ GA were excluded. Any definition of NE/HIE/PA that included PA or encephalopathy was eligible for inclusion.

Intervention: Only RCTs (or protocols for RCTs) of NE/HIE/PA interventions were eligible for inclusion in this systematic review. Any intervention for NE/HIE/PA was eligible for inclusion.

Comparator: Studies with any comparator to the intervention for NE/HIE/PA, including another treatment, placebo, standard care or nothing was eligible for inclusion.

Outcome: Studies that measured any outcome were eligible for inclusion.

Study design: Only RCTs (or protocols for RCTs) of NE/HIE/PA interventions were eligible for inclusion in this systematic review. Studies of other design types, such as case reports, case series, in vitro studies, or animal studies were excluded. Studies that reported clinical or biochemical eligibility criteria for diagnosis or inclusion in the RCT were extracted and included in the qualitative analysis.

Language: Only studies reported in English were eligible for inclusion.

### Screening and selection process

The literature search results were uploaded to Covidence software. Results were screened for inclusion against the selection criteria above by two of the four reviewers independently (FQ, DD, TH, AB), initially by screening titles and abstracts and subsequently screening the full text of the remaining studies. Disagreements were resolved by discussion (FQ, DD, TH, AB). Quality assessment was not completed as the purpose of the study was to describe variations in terminology, definitions, and eligibility criteria in RCTs for NE/HIE/PA and no meta-analysis was intended or performed.

### Outcome definition

The primary outcome was to describe the different terminology and definitions used for the condition, and to describe the types and frequency of different participant eligibility criteria for trials of treatments for NE/HIE.

### Data extraction

A data extraction form (DEF) was developed (TH, EM) and piloted on ten randomly selected studies. The DEF included the following domains: study descriptive data, terminology and definitions used, eligibility criteria employed, and study outcomes. Eligibility criteria were categorized as inclusion and exclusion criteria. Data from all included studies were extracted by two investigators independently (TH, AB, GK), and disagreements were resolved by consensus before data synthesis.

### Data synthesis

Categorical data were synthesized and are presented as counts, proportions and percentages. Grouping data into PA/neurological assessment categories was decided before data extraction. Where descriptive details were extracted from individual studies, this is presented directly. All quantitative analyses were conducted using SPSS software.

## Results

**Fig. 1:**
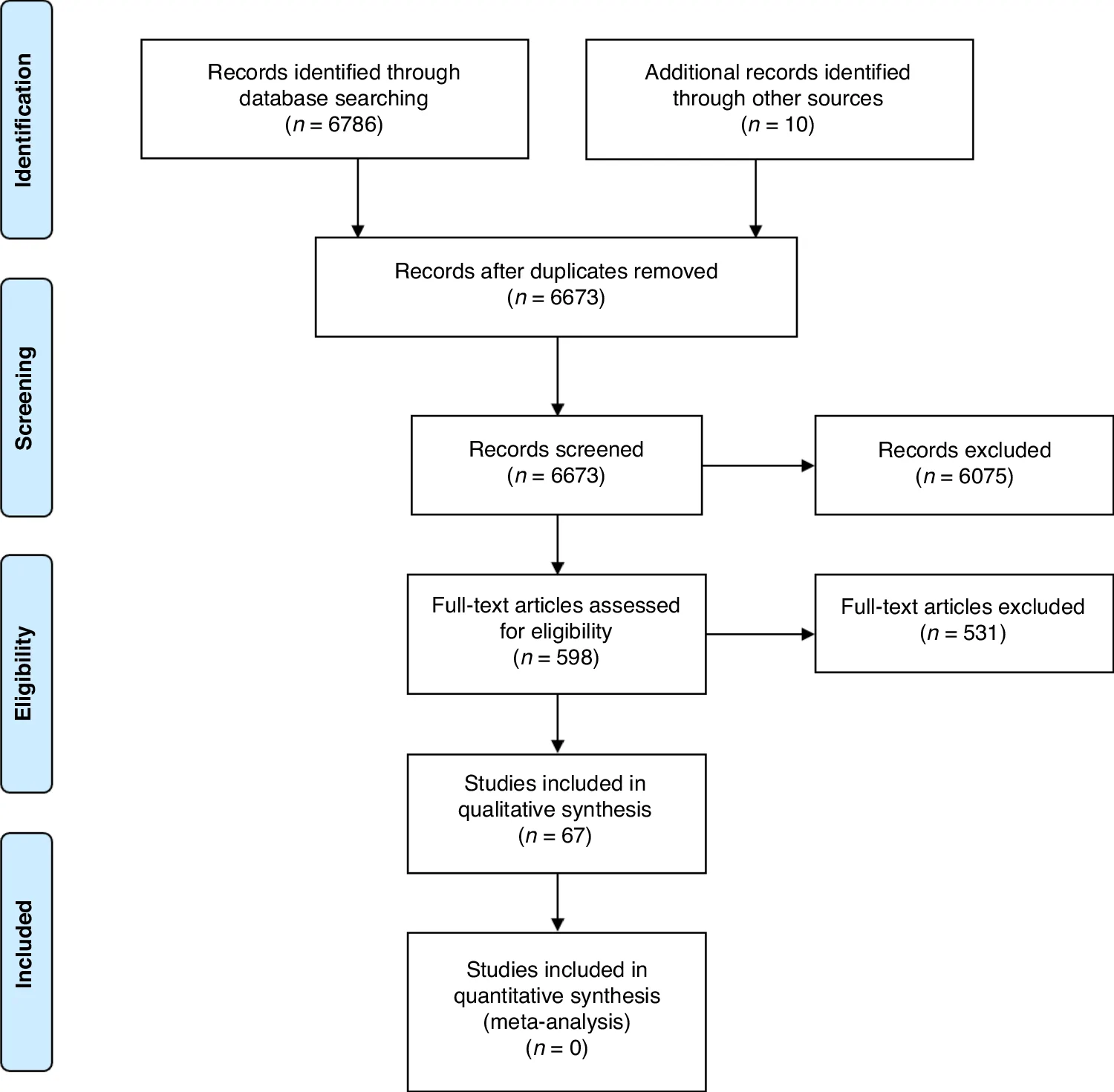
PRISMA flow diagram of study selection. Study selection flow diagram.

The literature search yielded 6786 results, and 10 further records were identified by searching the gray literature and the reference lists of retrieved articles. 6673 results remained following the exclusion of duplicates. We excluded 6075 records during title and abstract screening, leaving 598 for full text review and 67 studies were included in the data synthesis (Fig. 1).

RCTs were conducted in 20 different countries and published from 1998 to 2022. In total, 6412 participants were included in the 67 included studies. The effect of 16 different interventions including whole body TH, selective head cooling, TH plus inhaled xenon, erythropoietin (EPO), darbepoetin, melatonin, allopurinol, ascorbic acid and oral ibuprofen, magnesium sulfate, phenobarbital, pyritinol, fluid restriction, and early introduction of enteral feeds were examined in RCTs. All studies were conducted in a hospital setting.

### Terminology

**Fig. 2:**
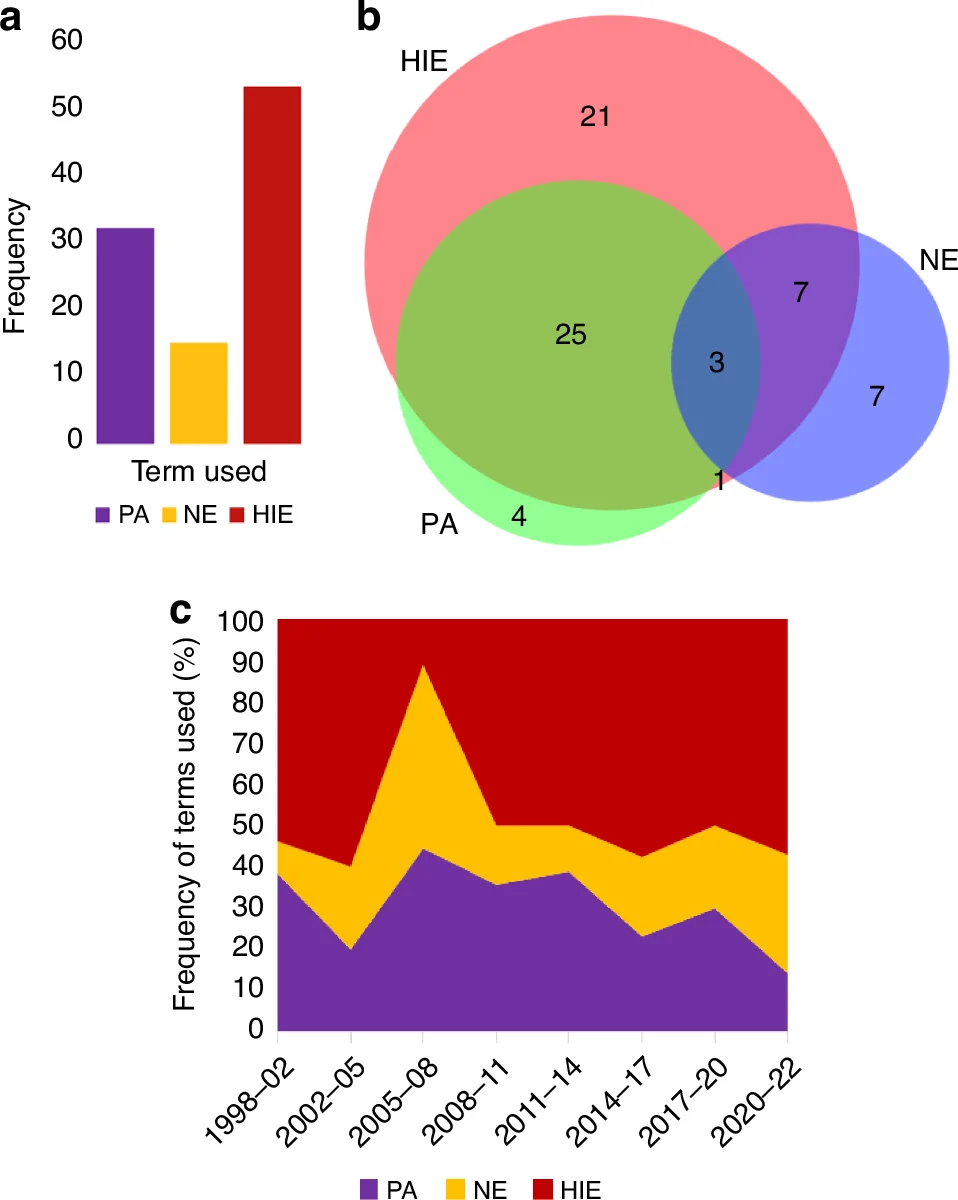
Terminology used in randomised controlled trials in PA/NE/HIE (*n* = 67). The frequency of terminology used in included studies (**a**), proportional Venn diagram of the frequency and overlap of terms used in included studies, created using BioVenn software (**b**), and trends in the frequency of terminology used from 1998 to 2022 (**c**). PA perinatal asphyxia, NE neonatal encephalopathy, HIE hypoxic ischaemic encephalopathy.

HIE was the most frequently used term to describe the condition (56/67 studies, 84%), then PA (33/67, 49%), and NE was the least frequently used (16/67 studies, 24%) (Fig. 2A)

Many of the papers used more than one term interchangeably. Most frequently, both HIE and PA were used in 25/67 studies (37%), followed by HIE and NE (7/67, 10%), with NE and PA used interchangeably in only 1 study (1%). Three studies (4%) used all the terms interchangeably (Fig. 2B).

### Terminology trends over time

HIE remained the most frequently used term to describe the condition, used in 50–60% of all studies from 1998 to 2022 except for a very short sharp decline to 1/9 (11%) from 2005 to 2008, but recovered immediately in the following period from 2008–2011 to >50% (Fig. 2C). PA ranged from a low frequency of use of 16% from 2020–2022 to a high frequency of use of 44% in 2008–2011. NE was initially increasing in frequency of use, rising from 8% (1998–2002) to 20% (2002–2005) and then to 44% (2005–2008) before decreasing again to a fluctuating frequency of between 11 and 33% from 2008 to 2022.

### Definitions

We extracted the definition used or referenced in each study. Nine studies defined the term used or referenced an existing definition of NE, HIE, or PA (Table 1) outside of the clinical criteria employed. All nine definitions were unique. Therefore, comparing the frequency of currently suggested definitions of NE/HIE/PA was impossible. Most studies initially discussed the prevalence and incidence of the condition and subsequently defined the condition by the participant eligibility criteria.

**Table 1:** Terminology and definitions reported in included studies.

Study	NE	HIE	PA	Definition
Ahmad 2018		+	+	WHO 2007: ‘the failure to initiate and sustain breathing at birth'
Aker 2019		+		Defined by clinical criteria
Akisu 2003		+	+	Defined by clinical criteria
Akula 2015	+			Defined by clinical criteria
Aly 2009		+		Defined by clinical criteria
Aly 2015		+	+	Defined by clinical criteria
Atici 2015		+		Defined by clinical criteria
Avasiloaiei 2013		+	+	Perinatal asphyxia occurs when an antenatal, intranatal or postpartum neurologic insult or any combination of the three leads to: (i) hypoxemia (decreased oxygen flow to the fetus/ newborn); (ii) hypercapnia (altered O_2_/CO_2_ exchange); and (iii) ischemia (inadequate perfusion of tissues and organs)
Azzopardi 2009			+	Defined by clinical criteria
Azzopardi 2013	+	+	+	Defined by clinical criteria
Baserga 2015	+	+		Defined by clinical criteria
Battin 2001		+	+	Defined by clinical criteria
Benders 2006		+	+	Defined by clinical criteria
Bharadwaj 2012		+	+	Defined by clinical criteria
Bhat 2009		+	+	Defined by clinical criteria
Catherine 2021		+		Defined by clinical criteria
Celik 2015		+		Defined by clinical criteria
Das 2017			+	Defined by clinical criteria
DuPont 2021	+			Defined by clinical criteria
Eicher 2005	+	+		Defined by clinical criteria
Farargy 2019		+		Defined by clinical criteria
Filipi 2017		+		Defined by clinical criteria
Gane 2014		+	+	Defined by clinical criteria
Gluckman 2005	+	+		Defined by clinical criteria
Groenendaal 2002		+	+	Defined by clinical criteria
Gunes 2007		+	+	Defined by clinical criteria
Gunn 1998		+	+	Defined by clinical criteria
Horn 2006		+		Hypoxic ischemic insults during labor [and the] brain injury that occurs in this way is an evolving process and the clinical manifestation of this injury is termed hypoxic ischemic encephalopathy'
Hu 2022		+		Defined by clinical criteria
Ichiba 2002		+	+	Defined by clinical criteria
Jacobs 2011		+		Defined by clinical criteria
Joy 2013		+	+	Defined by clinical criteria
Laptook 2017	+	+	+	Defined by clinical criteria
Li 2009		+	+	Defined by clinical criteria
Lin 2006		+	+	Defined by clinical criteria
Lv 2017	+	+		Group of Neonatology, Chinese Pediatric Society, Chinese Medical Association (2005) Diagnostic criteria for neonatal hypoxic-ischemic encephalopathy
Maiwald 2019	+	+	+	Defined by clinical criteria
Malla 2017		+	+	Defined by clinical criteria
Nair 2009			+	Defined by clinical criteria
Nunez-Ramiro 2019		+	+	Defined by clinical criteria
Prakash 2016		+	+	Defined by clinical criteria
Rahman 2015		+		Defined by clinical criteria
Rakesh 2017		+	+	Defined by clinical criteria
Robertson 2008	+		+	Defined by clinical criteria
Sami El Shimi 2014		+	+	Defined by clinical criteria
Shankaran 2002	+	+		Defined by clinical criteria
Shankaran 2005		+	+	Defined by clinical criteria
Shankaran 2014		+		Defined by clinical criteria
Shankaran 2017		+		Hypoxic-ischemic encephalopathy represents a subset of neonatal encephalopathy, defined by clinical criteria
Siddiqui 2021		+	+	need for neonatal resuscitation (rather than stabilization) at birth with APGAR scores (≤3 in 1 min and ≤7 in 5 min)'
Simbruner 2010	+			Defined by clinical criteria
Singh 2004		+		Defined by clinical criteria
Singh 2005		+		Defined by clinical criteria
Srinivasakumar 2015		+		Defined by clinical criteria
Sun 2012		+		Defined by clinical criteria
Tanigasalam 2015		+		Defined by clinical criteria
Tanigasalam 2018	+			Defined by clinical criteria
Thayyil 2013	+			Thompson encephalopathy score >5
Thayyil 2021	+			Neonatal encephalopathy–a condition arising from an unexpected lack of cerebral blood flow and oxygen supply to the fetal brain at the time of birth
Thoresen 2000		+		Defined by clinical criteria
Van Bel 1998		+	+	Defined by clinical criteria
van Rooij 2010		+		Defined by clinical criteria
Velaphi 2013		+	+	Defined by clinical criteria
Wu 2016	+	+		Defined by clinical criteria
Wu 2022		+		Neonatal hypoxic–ischemic encephalopathy refers to neurologic dysfunction resulting from a reduction of oxygen and blood flow to a fetus’s brain near the time of birth and is an important cause of brain injury in term and near-term infants
Zhou 2010	+	+		Defined by clinical criteria
Zhu 2009		+	+	Defined by clinical criteria

Only 9 studies defined the term used or referenced an existing definition of NE, HIE, or PA outside of the clinical criteria employed.

### Inclusion criteria

In trials of interventions for NE, participants frequently had to demonstrate evidence of PA first, followed by a neurological assessment to examine for evidence of neurological dysfunction. Participants needed to meet the inclusion criteria in both PA and neurological assessment categories to be included in trials. We therefore grouped the inclusion criteria into these two categories for this study.

### Perinatal asphyxia

**Fig. 3:**
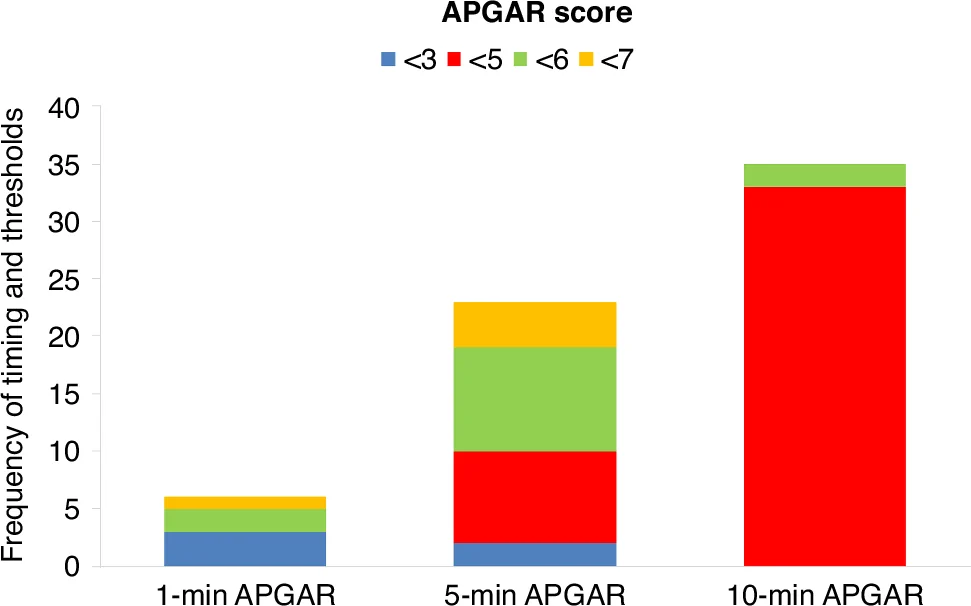
Inclusion criteria—Apgar score. Frequency of timing and threshold Apgar scores required for inclusion as a participant in trials for NE.

PA is defined as the impairment of gas exchange around the time before, during, or immediately after birth which, if prolonged, can lead to “progressive hypoxemia, hypercapnia, and significant metabolic acidosis^3^”. All included studies required postnatal evidence of PA which was a reduced Apgar score in the first few minutes after birth, evidence of metabolic acidosis on the umbilical cord blood gas or first postnatal gas, or the need for resuscitation in the period immediately after birth. The Apgar score was the most frequently used assessment of PA, used in 63 of 67 studies (94%) (Table 2). However, the timing and application of the Apgar score varied between studies. Assessment at 10 minutes was the most frequently employed, used in 35/67 studies (52%), assessment at 5 min was used in 23/67 studies (34%), and assessment at 1 min was only used in 6/67 studies (9%) (Fig. 3). Only three studies (4%) included assessment at multiple time points. A threshold score ≤5 was most frequently employed at 10 minutes, used in 33/67 studies (49%). However, there was much greater variability in threshold scores at 1- and 5-min assessments. At 5-min assessment, a score ≤5 was used in 8 of 67 studies (12%), a score ≤6 in 9/67 studies (13%), a score ≤7 in 4/67 studies (6%), and a score ≤3 in 2/67 studies (3%). At 1 min, a threshold score of ≤3 was used in 3/67 studies (4%), a score of ≤6 in 2/67 studies (3%) and ≤7 in 1/67 studies (1%).

**Table 2:** Frequency of inclusion criteria used as evidence of perinatal asphyxia for participants in trials for NE.

Study	Apgar	Acidosis	Resuscitation or ventilation
Ahmad 2018; Aker 2019	+	+	+
Akisu 2003	+	+	
Akula 2015	+	+	+
Aly 2009	+	+	
Aly 2015	+	+	
Atici 2015	+	+	+
Avasiloaiei 2013	+	+	
Azzopardi 2009	+	+	+
Azzopardi 2013	+	+	+
Baserga 2015	+	+	+
Battin 2001	+	+	
Benders 2006		+	+
Bharadwaj 2012	+	+	+
Bhat 2009	+	+	+
Catherine 2021	+	+	+
Celik 2015	+	+	+
Das 2017	+	+	+
DuPont 2021	+	+	+
Eicher 2005	+	+	+
Farargy 2019	+	+	
Filipi 2017	+	+	+
Gane 2014	+	+	+
Gluckman 2005	+	+	+
Groenendaal 2002	+	+	+
Gunes 2007	+	+	
Gunn 1998	+	+	
Horn 2006	+	+	+
Hu 2022	+		+
Ichiba 2002	+		+
Jacobs 2011	+	+	+
Joy 2013	+	+	+
Laptook 2017	+	+	+
Li 2009	+	+	+
Lin 2006	+	+	
Lv 2017	+	+	
Maiwald 2019	+	+	+
Malla 2017	+	+	+
Nair 2009 Nunez-Ramiro 2019	+	+	+
Prakash 2016	+		+
Rahman 2015	+	+	+
Rakesh 2017	+	+	+
Robertson 2008	+		+
Sami El Shimi 2014	+	+	+
Shankaran 2002	+	+	+
Shankaran 2005	+	+	+
Shankaran 2014	+	+	+
Shankaran 2017	+	+	+
Siddiqui 2021	+		+
Simbruner 2010	+	+	+
Singh 2004	+	+	
Singh 2005	+	+	
Srinivasakumar 2015	+	+	+
Sun 2012	+	+	+
Tanigasalam 2015	+	+	+
Tanigasalam 2018	+	+	+
Thayyil 2013 Thayyil 2021	+		+
Thoresen 2000	+	+	+
Van Bel 1998		+	+
van Rooij 2010	+	+	
Velaphi 2013	+	+	+
Wu 2016	+	+	+
Wu 2022	+	+	+
Zhou 2010	+	+	+
Zhu 2009	+		+

Apgar score was the most frequently used assessment of PA, used in 63 of 67 studies (94%), followed by metabolic acidosis on the umbilical cord blood gas or first postnatal blood gas, used in 58 of 67 studies (87%), and finally need for resuscitation or ventilation was used as a criterion in 52 of 67 studies (78%).

The next most frequently employed criterion used as evidence of PA was metabolic acidosis on the umbilical cord blood gas or first postnatal blood gas, used in 58/67 studies (87%) (Table 2). Both low pH and high base deficit (BD) were considered evidence of metabolic acidosis; however, low pH was employed in 55/67 studies (82%) compared to high base deficit (BD) which was used in 46/67 studies (69%). 43/67 studies (64%) accepted either criterion as evidence of metabolic acidosis.

**Fig. 4:**
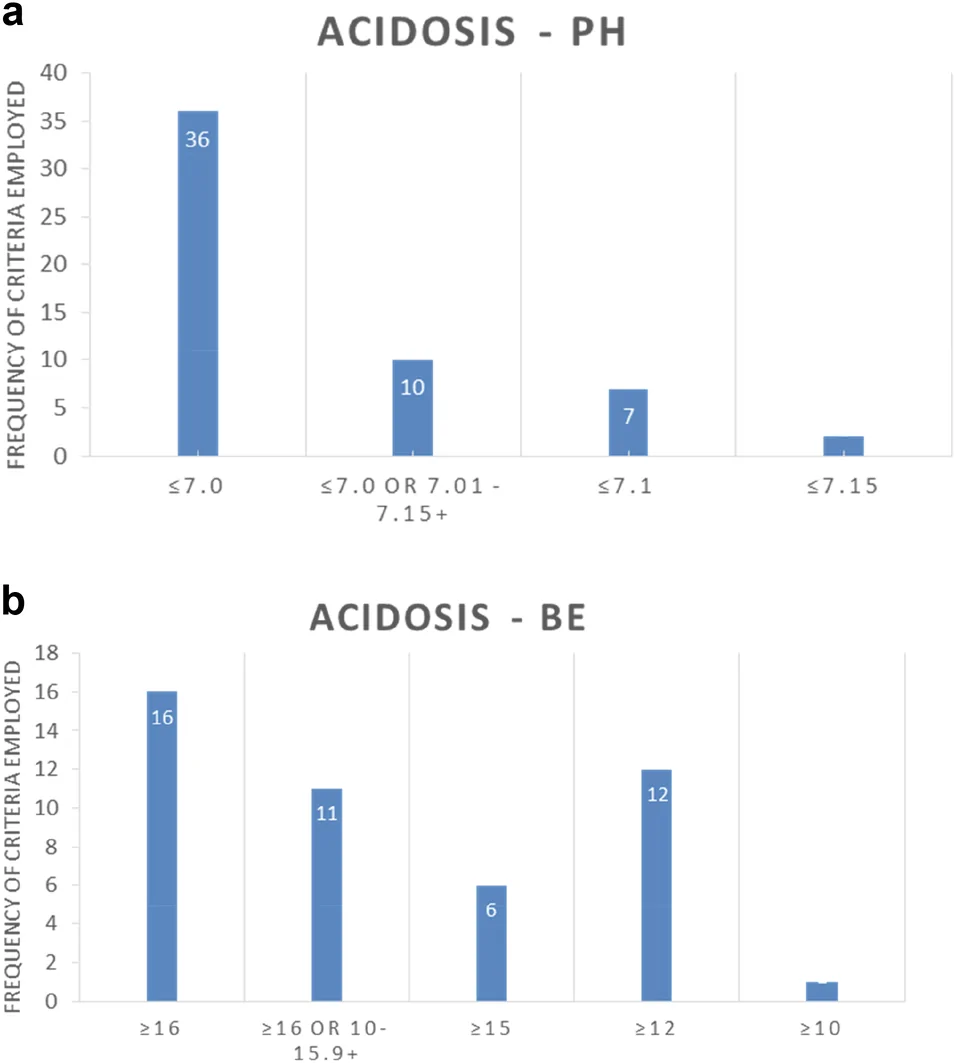
Inclusion criteria—evidence of metabolic acidosis. Frequency of threshold pH value required for inclusion as a participant in trials for PA/NE/HIE (**a**), and the frequency of threshold BE value required for inclusion as a participant in trials for PA/NE/HIE (**b**).

There was variation in the application of threshold scores. The most severe pH threshold score, ≤7.0, was the most frequently employed in 36/67 studies (54%) (Fig. 4A). A sole threshold pH of ≤7.0 or between 7.01 and 7.15 was acceptable if further criteria were met in 10/67 studies (15%). Participants with a pH ≤7.1 were eligible for inclusion in 7/67 studies (10%) and a pH <7.15 in 2/67 studies (3%).

The most severe BD threshold score, ≥16 mmol/L, was the most frequently employed in 16/67 studies (24%), and a score ≥15 mmol/L was used in 6/67 studies (9%), a score ≥12 mmol/L was used in 12/67 studies (18%), and 1 study used a threshold score of ≤10 mmol/L (1%) (Fig. 4B). Participants were eligible with a sole threshold BD of ≥16 mmol/L or between 10 and 15.9 mmol/L if further criteria were met in 11/67 studies (16%).

A need for resuscitation or ventilation was used as a criterion in 51 of 67 studies (76%) (Table 2). A need for resuscitation was used in all 51/67 studies (67%); however, ventilation was used as a criterion in 25/67 studies (37%).

### Neurological assessment

There were major differences in the application of neurological assessments between RCTs (Table 3). Several classifications and scoring systems have been developed for neurological assessment in the first few minutes and hours of life. The most frequently used include Sarnat staging,^22^ several modified versions of Sarnat staging, the Thompson score,^23^ and the neurological criteria from the NICHD-NRN^24^ or TOBY-BAPM TH studies.^25^ Very few RCTs explicitly referenced a classification or scoring system as the direct criterion for inclusion in an RCT; however, many referenced a system and extracted components to form their eligibility criteria.

**Table 3:** Summary of the neurological assessment in each RCT.

Study	Neurological assessment
Ahmad 2018	Thompson score used, no score specified for inclusion or exclusion
Aker 2019	NICHD 2005, Sarnat & Thompson for severity
Akisu 2003	Sarnat for severity - mild excluded
Akula 2015	Coolcap or NICHD criteria, Sarnat for severity
Aly 2009	Sarnat for severity
Aly 2015	Sarnat for severity - mild excluded
Atici 2015	ACOG 2004 criteria, modified Sarnat for severity - mild excluded
Avasiloaiei 2013	ACOG 2004 criteria, required ≥3 of 4 criteria
Azzopardi 2009	Own criteria (TOBY trial)
Azzopardi 2013	Own criteria, similar to TOBY trial but not referenced, Thompson severity score
Baserga 2015	NICHD 2005 trial criteria
Battin 2001	Own criteria
Benders 2006	Ongoing need for resus beyond 5 min, aEEG criteria
Bharadwaj 2012	Sarnat for severity - only moderate - severe included
Bhat 2009	Portman et al and Sarnat, Sarnat for severity
Catherine 2022	Sarnat - moderate or severe only included
Celik 2015	ACOG criteria, modified Sarnat for severity
Das 2017	Sarnat for severity - mild excluded
DuPont 2021	Modified Sarnat for severity, mild only, moderate or severe excluded
Eicher 2005	Sarnat for severity
Farargy 2019	Sarnat for severity - mild excluded
Filipi 2017	CRIB score - mild excluded
Gane 2014	“evidence of encephalopathy”, not specified further
Gluckman 2005	Sarnat - moderate or severe only included
Groenendaal 2002	Sarnat for severity, not specified for inclusion or exclusion
Gunes 2007	Sarnat for severity, not specified for inclusion or exclusion
Gunn 1998	“encephalopathy consisting of lethargy/stupor, hypotonia, abnormal reflexes including an absent or weak suc
Horn 2006	Thompson score ≥2
Hu 2022	Levene’s modification of Sarnat for severity - moderate or severe only
Ichiba 2002	“failure to initiate spontaneous respiration at 10 min after birth because of asphyxia, or the presence of clinically apparent seizures within 24 h after birth”
Jacobs 2011	Sarnat - moderate or severe only included
Joy 2013	Sarnat - moderate or severe only included
Laptook 2017	NICHD 2005 trial criteria
Li 2009	Sarnat - moderate or severe only included
Lin 2006	“clinical signs of postpartum encephalopathy (decreased muscle tone, lethargy, coma, or seizures) starting after birth”
Lv 2017	“diagnostic criteria of neonatal HIE formulated by the Chinese Medical Association”
Maiwald 2019	“must meet two out of the following four criteria for potentially evolving encephalopathy to participate in the study: (1) altered state of consciousness (reduced or absent response to stimulation or hyperexcitability); (2) severe hypotonia or hypertonia; (3) absent or insufficient spontaneous respiration (i.e., gasping only) with need for support at 10 min postnatally and/or (4) abnormal primitive reflexes (absent suck/gag/ corneal/Moro reflex) or abnormal movements (i.e., potential clinical correlates of seizure activity)”
Malla 2017	Modified Sarnat, moderate or severe only, required ≥3/6 criteria
Nair 2009	“clinical evidence of encephalopathy observed in the first 7 days of postnatal life” not specifed further
Nunez-Ramiro 2019	Modified Sarnat
Prakash 2016	Modified Sarnat
Rahman 2015	Reduced LOC + ≥ 1 of hypotonia, abnormal reflexes or absent or weak suck. Modified Sarnat - moderate or se
Rakesh 2017	Modified Sarnat
Robertson 2008	Thompson score >5
Sami El Shimi 2014	Thompson score, mild excluded
Shankaran 2002	Own, NICHD classification
Shankaran 2005	Own, NICHD classification
Shankaran 2014	NICHD 2005 trial criteria
Shankaran 2017	NICHD 2005 trial criteria
Siddiqui 2021	Sarnat for severity
Simbruner 2010	Own criteria from neo.nEURO.network
Singh 2004	“overt neurological syndrome in form of alteration of tone and/or sensorium within the first six hours of life” (Sarnat staging)
Singh 2005	“features of encephalopathy in the form of alterations of tone, deep tendon reflexes, primitive reflexes and sen (Sarnat staging)
Srinivasakumar 2015	NICHD criteria or seizures
Sun 2012	Sarnat (mild - severe)
Tanigasalam 2015	Sarnat criteria (mild - severe)
Tanigasalam 2018	Sarnat - moderate or severe only included
Thayyil 2013	Thompson score >5
Thayyil 2021	Modified Sarnat staging
Thoresen 2000	“Signs of encephalopathy, eg, lethargy, stupor, hypotonia, absent suck, and clinical seizures”
Van Bel 1998	Need for resusciation for >2 min, Sarnat for severity
van Rooij 2010	Sarnat for severity - mild excluded
Velaphi 2013	Sarnat for severity
Wu 2016	Sarnat for severity - moderate or severe only, mild excluded, req ≥3/6 sarnat criteria
Wu 2022	Modified Sarnat for severity - moderate or severe only, mild excluded
Zhou 2010	Sarnat (mild - severe)
Zhu 2009	Sarnat - moderate or severe only included

There is very wide variation in application of neurological exam criteria but the most frequently employed were Sarnat staging, several modified Sarnat staging, Thompson score, and the neurological criteria from the NICHD or TOBY therapeutic hypothermia studies.

StudyLOC Posture Tone Abnormal reflexes Weak suck Seizures Autonomic dysfn aEEG EEG.

The most frequently used criterion from the neurological assessment was a reduced level of consciousness, included in 57 of 67 studies (85%), followed by reduced tone included in 55 studies (82%), abnormal reflexes in 54 studies (81%), and weak suck in 51 studies (76%) (Table 4). Less frequently employed criteria included abnormal posture, used in 43 studies (64%), and autonomic dysfunction in 41 studies (61%).

**Table 4:** Frequency of inclusion criteria used as evidence of neurological dysfunction for participants in trials for NE.

Ahmad 2018	+	+	+	+	+	+	+	-	-
Aker 2019	+	+	+	+	+	+	+	-	-
Akisu 2003	+	-	+	+	-	-	-	-	-
Akula 2015	+	+	+	+	+	+	+	-	-
Aly 2009	+	-	+	-	-	+	-	-	-
Aly 2015	+	+	+	+	+	+	-	-	-
Atici 2015	+	+	+	+	-	+	+	+	-
Avasiloaiei 2013	-	-	-	-	-	-	-	-	-
Azzopardi 2009	+	+	+	+	+	+	-	+	-
Azzopardi 2013	+	+	+	+	+	+	-	+	-
Baserga 2015	+	+	+	+	+	+	+	-	-
Battin 2001	+	+	+	+	+	-	-	-	-
Benders 2006	-	-	-	-	-	+	-	+	-
Bharadwaj 2012	+	+	+	+	+	+	+	-	-
Bhat 2009	+	+	+	+	+	-	+	-	-
Catherine 2021	+	+	+	+	+	+	+	-	-
Celik 2015	+	+	+	+	+	+	+	+	-
Das 2017	+	-	+	+	+	+	-	-	-
DuPont 2021	+	+	+	+	+	+	+	-	-
Eicher 2005	+	+	+	+	-	+	+	-	-
Farargy 2019	+	-	+	+	+	-	-	-	-
Filipi 2017	+	-	+	+	+	+	-	+	-
Gane 2014	-	-	-	-	-	-	-	-	-
Gluckman 2005	+	+	+	+	+	+	+	+	-
Groenendaal 2002	-	-	-	-	-	-	-	-	-
Gunes 2007	+	-	+	+	+	+	-	-	-
Gunn 1998	+	-	+	+	+	-	-	-	-
Horn 2006	+	+	+	+	+	+	+	-	-
Hu 2022	+	+	+	+	+	+	+	-	-
Ichiba 2002	-	-	-	-	-	+	-	-	-
Jacobs 2011	+	+	+	+	+	+	+	-	-
Joy 2013	+	+	+	+	+	+	+	-	-
Laptook 2017	+	+	+	+	+	+	+	-	-
Li 2009	+	+	+	+	+	-	+	-	-
Lin 2006	+	-	+	-	-	+	-	-	-
Lv 2017	+	-	+	+	+	+	+	+	-
Maiwald 2019	+	+	+	+	+	+	+	-	-
Malla 2017	+	+	+	+	+	-	+	-	-
Nair 2009	-	-	-	-	-	-	-	-	-
Nunez-Ramiro 2019	+	+	+	+	+	-	+	-	-
Prakash 2016	-	-	-	-	-	-	-	-	-
Rahman 2015	+	-	-	+	+	+	+	+	-
Rakesh 2017	+	+	+	+	+	-	+	-	-
Robertson 2008	+	+	+	+	+	-	+	-	-
Sami El Shimi 2014	+	+	+	+	+	+	+	-	-
Shankaran 2002	+	+	+	+	+	+	+	-	-
Shankaran 2005	+	+	+	+	+	+	+	-	-
Shankaran 2014	+	+	+	+	+	+	+	-	-
Shankaran 2017	+	+	+	+	+	+	+	-	-
Siddiqui 2021	+	+	+	+	+	+	+	-	-
Simbruner 2010	+	-	+	+	+	+	+	+	+
Singh 2004	+	-	-	-	-	-	-	-	-
Singh 2005	+	-	+	+	+	-	-	-	-
Srinivasakumar 2015	+	+	+	+	+	+	+	-	-
Sun 2012	+	+	+	+	+	-	+	-	-
Tanigasalam 2015	+	+	+	+	+	-	+	-	-
Tanigasalam 2018	+	+	+	+	+	-	+	-	-
Thayyil 2013	+	+	+	+	+	+	-	-	-
Thayyil 2021	+	+	+	+	+	-	+	-	-
Thoresen 2000	+	-	+	+	+	+	-	+	+
Van Bel 1998	-	-	-	-	-	-	-	-	-
van Rooij 2010	-	-	-	-	-	+	-	-	-
Velaphi 2013	-	-	-	-	-	-	-	-	-
Wu 2016	+	+	+	+	+	+	+	-	-
Wu 2022	+	+	+	+	+	-	+	-	-
Zhou 2010	+	+	+	+	+	+	+	-	-
Zhu 2009	+	+	+	+	+	+	+	-	-

The most frequently used criterion was reduced level of consciousness (85%), followed by reduced tone (82%), abnormal reflexes (81%), and weak suck (76%). Less frequently employed criteria included abnormal posture (64%), autonomic dysfunction (61%), and aEEG (16%).

### Other eligibility criteria

Several studies employed additional eligibility criteria outside of postnatal evidence of PA and neurological dysfunction on clinical examination. The most frequently used was evidence of a perinatal sentinel event, an acute event in the perinatal history that possibly compromises placental blood flow,^26^ which was used in 29 of 67 studies (43%). Electrophysiological monitoring by electroencephalography (EEG) or amplitude-integrated EEG (aEEG) to detect abnormal cerebral function was used in 2 of 67 (3%) and 11 of 67 studies (16%) respectively. There was significant variation in reporting of EEG between studies: many used a general description of abnormal background activity and others specific patterns like burst suppression. Evidence of multi-organ dysfunction was employed as a criterion in 9 of 67 studies (13%).

### Exclusion criteria

Gestational age was the most frequently employed exclusion criterion, used in 63/67 studies (94%), followed by the presence of major congenital abnormalities in 62/67 studies (93%), and the need for identification and diagnosis within 6 h of birth, used in 52/67 studies (78%) (Table 5). Participants with mild NE/HIE/PA were excluded in 35 of 67 studies (52%). Participants with low birthweight were excluded in 29 of 67 studies (43%) and participants born small for gestational age were excluded in 20 of 67 studies (30%). Participants with neonatal sepsis were excluded in 12 of 67 studies (18%). There was significant variation in the use of the term sepsis without further explanation and others mentioned a clinical diagnosis of sepsis. Only one included a definition of blood, urine, or CSF positive culture plus clinical signs of sepsis.^27^

**Table 5:** Frequency of exclusion criteria used for participants in trials for NE.

Study	Gestational Age Birth weight Major CA			IUGR or SGA		Sepsis	Mild NE	Enroll 6 h
Ahmad 2018	+	-	-	-	-		-	-
Aker 2019	+	+	+	-	-		+	+
Akisu 2003	+	-	+	-	-		+	+
Akula 2015	+	+	+	+	-		+	+
Aly 2009	+	-	+	-	+		-	-
Aly 2015	+	+	+	+	+		+	+
Atici 2015	+	-	+	+	-		+	+
Avasiloaiei 2013	+	-	+	-	-		+	-
Azzopardi 2009	+	-	+	-	-		+	+
Azzopardi 2013	+	-	+	-	-		+	+
Baserga 2015	+	+	+	+	-		+	+
Battin 2001	+	-	+	-	-		-	+
Benders 2006	+	-	+	-	-		-	+
Bharadwaj 2012	+	-	+	-	-		+	+
Bhat 2009	+	-	+	+	-		+	+
Catherine 2021	+	-	+	-	-		+	+
Celik 2015	+	-	+	+	-		-	+
Das 2017	+	+	+	+	-		+	+
DuPont 2021	+	-	+	+	-		-	-
Eicher 2005	+	+	+	+	+		-	-
Farargy 2019	-	-	+	+	+		+	-
Filipi 2017	+	+	+	-	-		+	-
Gane 2014	+	-	+	-	-		-	+
Gluckman 2005	+	+	+	+	-		+	+
Groenendaal 2002	+	-	-	-	-		-	+
Gunes 2007	+	-	+	-	+		-	-
Gunn 1998	+	-	+	-	-		-	+
Horn 2006	+	-	+	-	+		-	-
Hu 2022	+	+	+	-	-		+	+
Ichiba 2002	+	-	+	-	+		-	-
Jacobs 2011	+	+	+	-	-		+	+
Joy 2013	+	-	+	-	-		+	+
Laptook 2017	+	+	+	+	-		+	+
Li 2009	+	+	+	-	-		+	-
Lin 2006	+	-	+	-	-		-	+
Lv 2017	+	+	+	-	+		+	-
Maiwald 2019	+	+	+	-	-		+	+
Malla 2017	+	-	+	+	-		+	+
Nair 2009	+	-	+	-	+		-	-
Nunez-Ramiro 2019	+	+	-	-	-		-	+
Prakash 2016	-	-	+	-	-		-	+
Rahman 2015	+	-	+	-	-		+	+
Rakesh 2017	+	-	+	-	-		-	+
Robertson 2008	+	+	-	+	-		-	+
Sami El Shimi 2014	+	+	+	+	+		+	+
Shankaran 2002	+	+	+	+	-		+	+
Shankaran 2005	+	+	+	+	-		+	+
Shankaran 2014	+	+	+	+	-		+	+
Shankaran 2017	+	+	+	+	-		+	+
Siddiqui 2021	-	-	+	-	-		-	+
Simbruner 2010	+	+	+	-	-		+	+
Singh 2004	+	-	+	-	-		-	+
Singh 2005	+	-	+	-	-		-	+
Srinivasakumar 2015	+	-	+	+	-		+	+
Sun 2012	+	+	+	-	+		-	+
Tanigasalam 2015	+	-	+	-	-		-	+
Tanigasalam 2018	+	-	+	-	-		+	+
Thayyil 2013	-	-	-	-	-		-	+
Thayyil 2021	+	+	+	-	-		+	+
Thoresen 2000	+	-	+	-	-		+	+
Van Bel 1998	+	-	+	-	-		-	+
van Rooij 2010	+	-	+	-	-		-	-
Velaphi 2013	+	+	+	-	-		-	+
Wu 2016	+	+	+	-	-		+	+
Wu 2022	+	+	+	-	-		+	+
Zhou 2010	+	+	+	-	+		-	+
Zhu 2009	+	+	+	-	-		+	-

Gestational age was the most frequently employed exclusion criteria (94%), followed by the presence of major congenital abnormalities (93%), and the need for identification and diagnosis within 6 h of birth (78%).

**Fig. 5:**
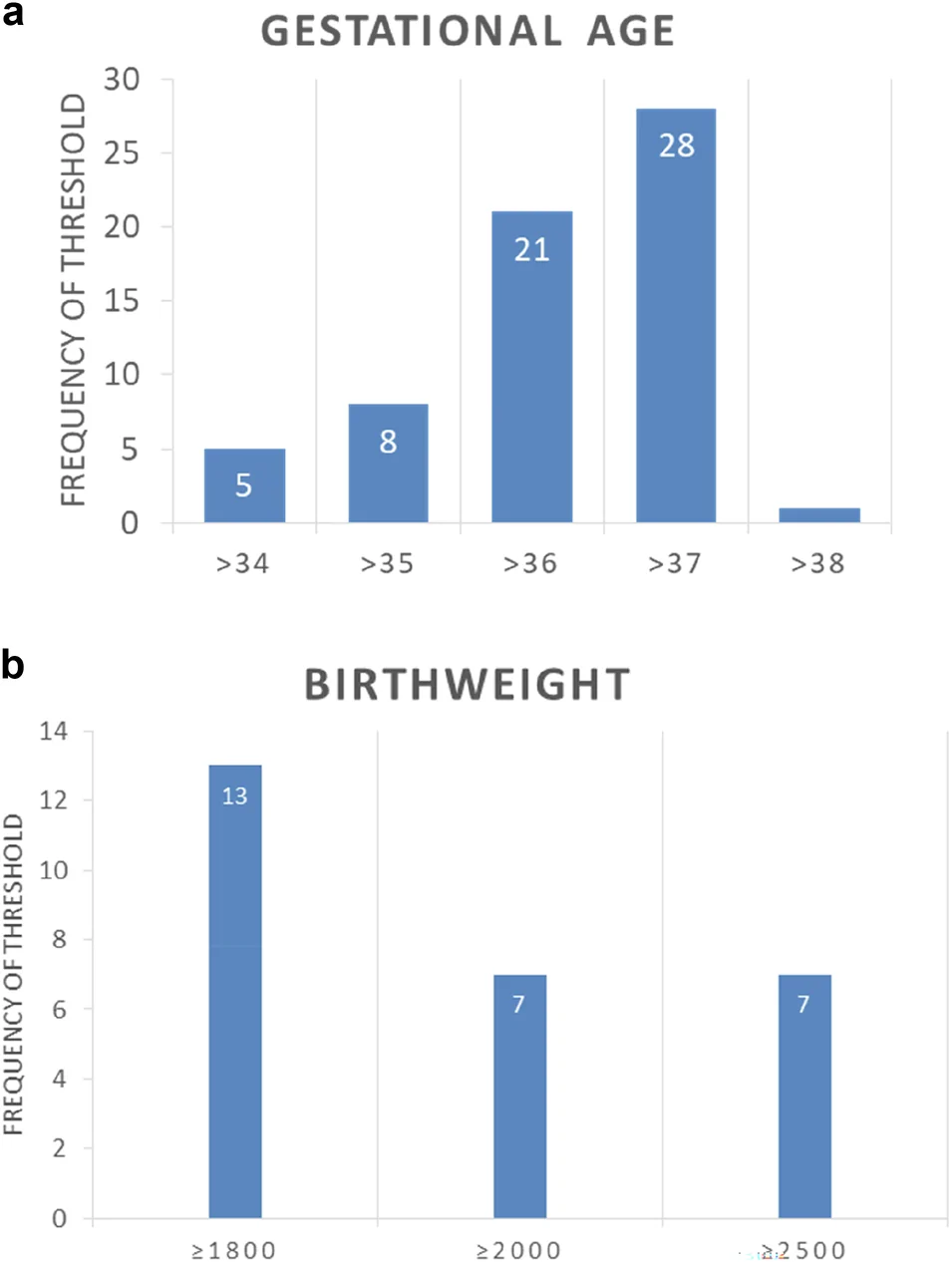
Exclusion criteria—gestational age and birthweight. Frequency of threshold gestational age in weeks required for exclusion as a participant in trials for PA/NE/HIE (**a**) and the frequency of threshold birthweight in grams required for exclusion as a participant in trials for PA/NE/HIE (**b**).

Of the 63 studies that employed gestational age as an exclusion criterion, term gestation of ≥37 completed weeks’ gestational age at birth was the most frequently employed threshold for participation in RCT (28/63 studies (44%)), followed by late preterm gestational ages at birth of ≥36 weeks ((21/63 (33%) and ≥35 weeks (8/63 (13%)) (Fig. 5A). 5/63 (8%) studies used a GA threshold of ≥34 weeks at birth and 1/63 (2%) used a GA threshold ≥38 weeks at birth.

The most frequently employed minimum birthweight threshold employed was ≥1800 g, used in 15 of 29 studies (52%), followed by a threshold of ≥2500 g and a threshold of ≥2000 g which were both used in 7 of 29 studies (24%) (Fig. 5B).

As the only intervention widely available for patients with NE/HIE/PA in high-resource settings, we selected the large trials of TH with >100 participants to compare their eligibility criteria and control group mortality (Table 6). These trials provide the eligibility criteria for TH in many national guidelines in patients with NE/HIE/PA.^13,14^ Studies were in universal agreement regarding the Apgar score threshold of ≤5 at 10 min of life. Three studies required evidence of metabolic acidosis on cord blood gas or first postnatal blood gas of a pH value of ≤7.0, and one study accepted a pH of 7.01–7.15 mmol/L if further criteria were met. Two studies required a BD of ≤ −16mmol/L, one a BD of ≤ −12mmol/L, and one accepted a BD of −10 to −15.9 mmol/L if further criteria were met. Studies were in universal agreement regarding the use of the need for resuscitation at 10 min of life as an inclusion criterion. However, two of the studies specified the need for ventilation as part of the resuscitation at 10 min of life. Only one study included perinatal sentinel events as an inclusion criterion. All studies were in universal agreement regarding the use of modified Sarnat staging as the neurological assessment for inclusion in trials for NE. The application of modified Sarnat staging differed significantly between studies as follows: any staging accepted (*n* = 1), moderate-severe encephalopathy (*n* = 1), reduced level of consciousness with one further sign (*n* = 1) and signs in three categories (*n* = 1). Two studies included abnormalities on aEEG as a criterion for inclusion. Studies were similar in the threshold for exclusion based on gestational age at birth, three using ≤36 weeks and one using ≤35 weeks, and in the threshold for exclusion based on birthweight, two using ≤1800 g and one using ≤2000 g. All studies excluded patients with major congenital abnormalities. All studies excluded patients not enrolled within 6 h of birth.

**Table 6:** Eligibility criteria and control group mortality in large trials of therapeutic hypothermia for neonatal encephalopathy.

Criteria	Coolcap	NICHD	TOBY
Apgar	10 ≤ 5	10 ≤ 5	10 ≤ 5
Acidosis	pH < 7.0 or BE ≤ −16	pH < 7.0/7.01–7.15 or BE ≤ −16/−10–15.9(+)	pH < 7.0 or BE ≤ −16
Resuscitation	≥10 min	Ventilation ≥ 10 min	≥10 min
PSE	No	Yes	No
Neurological	Sarnat – mod/severe only	1+ signs in ≥ 3/6 categories	Altered LoC + 1 other feature
aEEG	Abnormal background or seizures	No	Abnormal background or seizures
GA	≥36	≥36	≥36
Birthweight	<1800 g	<1800 g	N/A
MCA	Yes	Yes	Yes
Enroll by 6 h	Yes	Yes	Yes
Mortality (%)	38	27	27

*PSE* perinatal sentinel event, *aEEG* Amplitude-integrated electroencephalography, *GA* gestational age, *LoC* Level of Consciousness, *MCA* major congenital abnormality, *BE* base excess.

### Control group mortality

To assess the effect of differences in case definition criteria on trial participants included, we compared the control group mortality across large trials of TH. Control group mortality varied significantly between included studies, from the lowest rate of 2% to the highest rate of 67% (Table 7). The control group was treated with TH in 16 of 60 studies (27%). The control group mortality in those treated with TH was substantially different compared to the entire cohort, with the lowest mortality rate of 0% and the highest mortality rate of 33%. The duration of follow up varied substantially from a follow-up duration of 3 days to 24 months.

**Table 7:** Intervention, follow up duration, and control group mortality in interventional trials for NE.

Study	Year	*n*=	Intervention	Control TH	Control Mortality *n* (%)	Duration of follow up
Ahmad	2018	80	Melatonin	No	14 (35)	Death prior to hospital discharge
Aker	2019	50	TH	No	1 (4)	Death prior to hospital discharge
Akisu	2003	21	TH (SHC)	No	2 (20)	Death within the first 3 days of life
Akula	2015	100	TH (Device in transport)	Yes	6 (12)	Death prior to hospital discharge
Aly	2009	60	Ascorbic acid and ibuprofen	No	10 (33)	Not specified
Aly	2015	30	Melatonin	Yes	4 (27)	Death within the first 6 months of life
Atici	2015	30	SHC vs WBC	Yes	1 (8)	Death prior to hospital discharge (Control group considered WBC for this study)
Avasiloaiei	2013	67	Phenobarbitol or EPO	No	4 (17)	Not specified - participants followed up to 18 months
Azzopardi	2009	325	TH	No	44 (27)	Not specified - participants followed up to 18 months
Azzopardi	2016	92	Xenon	Yes	9 (20)	Death prior to hospital discharge
Baserga	2015	30	Darbepoetin	Yes	1 (10)	Death within the first 7 days of life
Battin	2001	40	SHC	No	3 (15)	Death within the first 7 days of life (‘Early neontal death’)
Benders	2006	32	Allopurinol	No	10 (67)	Death prior to hospital discharge
Bharadwaj	2012	124	TH (Gel packs)	No	6 (10)	Death prior to hospital discharge
Bhat	2009	40	Magnesium sulfate	No	2 (10)	Death prior to hospital discharge
Catherine	2021	162	TH	Yes	29 (35)	Death prior to hospital discharge
Celik	2015	31	SHC vs WBC	N/A	4 (33)	Death within the first 12 months of life
Das	2017	60	TH	No	9 (30)	Death within the first 30 months of life
DuPont	2021	21	Darbepoetin	No	0 (0)	Not specified - participants followed up to 12 months
Eicher	2005	65	TH	No	14 (42)	Death within the first 12 months of life
Farargy	2019	60	Magnesium sulfate & melatonin vs melatonin alone	Yes	N/A	Neither group considered to have standard therapy
Filipi	2017	44	Topiramate	Yes	2 (9)	Death within the first 24 months of life
Gane	2014	122	TH (Gel packs)	No	8 (16)	Death within the first 12 months of life
Gluckman	2005	234	SHC	No	42 (38)	Death within the first 18 months of life
Groenendaal	2002	22	Magnesium sulfate	No	6 (43)	Death within the first 24 months of life
Gunes	2007	60	Allopurinol	No	3 (11)	Death within the first 12 months of life
Gunn	1998	22	SHC	No	2 (20)%	Death within the first 18 months of life
Horn	2006	20	SHC (solid ice cap)	No	Not reported	N/A
Hu	2022	92	Early vs delayed enteral nutrition	Yes	N/A	N/A
Ichiba	2002	34	Magnesium sulfate	No	1 (6)	Death within the first 14 days of life
Jacobs	2011	221	TH	No	42 (38)	Death within the first 24 months of life
Joy	2013	116	TH	No	4 (7)	Death prior to hospital discharge
Laptook	2017	168	TH (beyond 6 h of life)	No	5 (6)	Death within the first 22 months of life
Li	2009	93	TH (initiated within the first 10 h of life)	No	3 (7)	Death within the first 18 months of life
Lin	2006	62	TH (SHC)	No	2 (7)	Death within the first 10 days of life
Lv	2017	42	EPO	Yes	0 (0)	Death within the first 9 months of life
Maiwald	2019 N/A	Allopurinol	Yes	N/A	Protocol only	
Malla	2017	100	EPO	No	8 (16)	Death within the first 19 months of life
Nair	2009	108	Pyritinol	No	1 (2)	Death within the first 12 months of life
Nunez-Ramiro	2019	180	Topiramate	Yes	10 (19)	Death prior to hospital discharge
Prakash	2016	120	Magnesium sulfate	No	Not reported	Only a composite of death or disability reported
Rahman	2015	60	Magnesium sulfate	Yes	5 (16)	Death prior to hospital discharge
Rakesh	2017	120	TH	No	16 (28)	Death within the first 6 months of life
Robertson	2008	36	TH	No	1 (7)	Death prior to hospital discharge
Sami El Shimi	2014	30	EPO	Yes	2 (20)	Death within the first 3 months of life
Shankaran	2002	19	TH	No	3 (30)	Death prior to hospital discharge
Shankaran	2005	239	TH	No	29 (27)	Death within the first 22 months of life
Shankaran	2014	364	TH (2 × 2 comparison of longer (120 h) and deeper (32) TH)	Yes	7 (7)	Death within the first 22 months of life
Shankaran	2017	364	TH (2 × 2 comparison of longer (120 h) and deeper (32) TH)	Yes	7 (7)	Death within the first 22 months of life
Siddiqui	2021	82	Magnesium sulfate	No	14 (35)	Death prior to hospital discharge
Simbruner	2010	129	TH	No	33 (57)	Death within the first 21 months of life
Singh	2004	45	Phenobarbital	No	3 (15)	Death prior to hospital discharge
Singh	2005	45	Phenobarbital	No	3 (15)	Death prior to hospital discharge
Srinivasakumar	2015	72	Treatment of electrographic seizures vs clincial seizures only	Yes (some) 2 (6)		Death within the first 24 months of life
Sun	2012	56	SHC	No	1 (4)	Death prior to hospital discharge
Tanigasalam	2015	120	TH	No	30 (50)	Death prior to hospital discharge
Tanigasalam	2018	80	Fluid restriction	Yes	3 (8)	Death within the first 6 months of life
Thayyil	2013	33	TH	No	2 (13)	Death prior to hospital discharge
Thayyil	2021	408	TH	No	63 (32)	Death within the first 22 months of life
Thoresen	2000	9	TH (intervention study but not a RCT)	No	Not reported	N/A
Van Bel	1998	22	Allopurinol	No	6 (55)	Death prior to hospital discharge
van Rooij	2010	42	Treatment of aEEG seizures	No	7 (50)	Death in the neonatal period
Velaphi	2013	94	Phenobarbital	No	7 (16)	Death prior to hospital discharge
Wu	2016	50	EPO	Yes	5 (19)	Death prior to hospital discharge
Wu	2022	501	EPO	Yes	28 (12)%	Death within the first 22–36 months of life
Zhou	2010	256	TH (SHC)	No	27 (29)	Death within the first 18 months of life
Zhu	2009	167	EPO	No	4 (5)	Death within the first 18 months of life

## Discussion

HIE is a subset of NE and has a complex pathophysiology, a broad spectrum of severity, and only one specific well-evidenced intervention which requires very early identification for treatment to be initiated, often before many diagnostic test results are available. It is also much more prevalent in resource-limited settings with limited access to diagnostic tests and where the intervention, TH, is often not feasible and not considered safe or beneficial.^28,29^ All these features mean it is difficult to agree on a universal definition and the criteria required for diagnosis.

Despite recommendations suggesting the use of the term NE, and for HIE to be reserved for a cause-specific subgroup of those with NE, HIE remains the most prevalent term used to describe the condition in trials of interventions for HIE. There is no evidence that the ACOG/AAP Task Force on NE and Neurologic Outcome^3^ definition of NE and the recommendation for the use of the term NE has been universally accepted, and the trend remains to use the term HIE. Since the ACOG/AAP Taskforce has recently defined NE and HIE specifically, it may become more challenging to identify when authors intend to use NE rather than HIE and when HIE, by the Task Force’s definition, is being used deliberately. This has implications for understanding the epidemiology of the condition, which, to date, has described separate entities with the incidence of NE estimated at 3.0 per 1000 births and the incidence of HIE at 1.5 per 1000 births.^30,31^ There is no universal implementation of the definitions provided by the Taskforce (Freeman, 2008) it is of note that the definition group was primarily composed of clinicians, and therefore does not reflect the input and thoughts of patients, parents, caregivers, families, and researchers in this field. Therefore, there must be a universal understanding of what is meant by the terms NE, HIE and PA, and that use of the terms is commonly understood. A universal understanding and definition of the meaning of NE/HIE/PA would provide diagnostic clarity, improved estimation of incidence, and improved identification of risk factors. This would also improve the clarity of the eligibility criteria for inclusion in future trials of interventions for HIE.

The Apgar score comprises a clinical assessment of five components: color, heart rate, reflexes, muscle tone, and breathing, and has advantages due to its immediate availability ease of use, and no requirement for measurement of equipment.^32^ This may be one reason it is the most consistently used inclusion criterion for trials in NE, used in 92% of all studies. A threshold of a score ≤5 at 10 min was most frequently employed; however, there was substantial variation in the application of timing of assessment and threshold values for inclusion. There is variable evidence of the discriminatory value of the Apgar score. The NICHD trial found that 65% of those with an Apgar score <5 had died or survived with disability compared to 30% of participants with a score ≥5.^33^ A large population-based cohort study demonstrated that increased mortality among anoxic term patients with a 5-min Apgar score of 4–6 (mortality rate of 54/1000) compared to an Apgar score of 7–10 (mortality rate of 0.42/1000),^34^ while another demonstrated a stronger association between later diagnosis of cerebral palsy and 10-minute Apgar score than 5-min Apgar score.^35^ ACOG and AAP caution, however, that the Apgar score cannot be used as a consequence of, or evidence for asphyxia as many neonatal conditions may cause a decrease in Apgar score and it is not predictive of individual risk of mortality or neurologic outcome.

Selecting only the most severe cases with evidence of PA may fail to provide TH to patients who may benefit from the treatment, and the evidence regarding the use of TH for those with mild NE/HIE/PA remains uncertain.^18^ Nelson et al. demonstrated from the Vermont Oxford Network NE Registry that only half of the eligible patients had a pH < 7.09 or a BD ≤ −12mmol/L.^36^ Therefore, the population meeting eligibility criteria for TH currently does not reflect the RCT trial eligibility criteria, and the RCT results may not be generalizable to the population outside of the eligibility criteria. Furthermore, if the intention is to identify patients at risk of adverse outcomes, only those eligibility criteria associated with increased risk of adverse outcomes should be included. Threshold values of pH, BD, and Apgar score should reflect current evidence of which values are associated with increased risk of adverse outcome and not only on expert consensus or clinical trial eligibility criteria. We found that while evidence of acidosis was a frequently employed eligibility criterion, there was wide variation in the application of pH threshold values and even broader variation in BD threshold values, with the most severe of each being the most frequently employed. A large systematic review before the introduction of routine treatment with TH demonstrated a strong temporal association between low cord pH and increased neonatal mortality, HIE, and cerebral palsy.^37^ A recent study suggests that an umbilical arterial cord blood gas pH of <7.10 has a sensitivity of 74% and a specificity of 99%. However, if a threshold value of <7.0 had been employed, 34 of the 69 patients treated with TH would not have been eligible.^38^ The study estimates that 25 neonates with a pH 7.0–7.1 would require screening to identify one neonate with moderate-severe encephalopathy, resulting in a 15% increase in appropriate selection for TH compared to a threshold value of <7.0. Other recent studies have demonstrated a dose-dependent relationship between base deficit and death or cerebral palsy, using an initial threshold value of < −12mmol/L.^39^ The strength of association with BD < −12mmol/L is unclear. Furthermore, if there is to be a universal definition of NE/HIE/PA and eligibility criteria for future trials, this will have to account for differences in local resources for measurement of biochemical parameters as evidence of metabolic acidosis may not always be available.

We found significant variation in criteria employed for assessing neurological dysfunction and minimal consistency in the use of standardized systems. In contrast to the NICHD system, Sarnat staging and Thompson scores, and variations of these systems, were developed for prediction rather than the classification of severity.^40^ There is significant subjectivity regarding the assessment of many aspects of neurological dysfunction. Among the most frequently employed neurological criteria were reduced level of consciousness, reduced tone, and abnormal reflexes, which were used in over 75% of included studies. A recent study demonstrated that although there were high levels of inter-observer agreement on the neurological status of a newborn when standardized NE/HIE/PA exam criteria were applied, differences in the application of these tests resulted in significant differences in the proportion of newborns eligible for TH.^19^ Both subjectivity in neurologic assessment and variability in neurologic exam application to TH eligibility were identified by the Society for Pediatric Research (SPR). SPR have recommended standardization of the neurological exam, training and validation of each examiner, and consistent timing of serial neurological exams for future neuroprotective trials in NE/HIE/PA.^20^

Gestational age (GA) was an exclusion criterion almost universally applied (97%). Threshold values of GA ranged from ≥36 or ≥37 weeks, accounting for 80% of all included studies, and only 20% used a threshold GA of ≥34 or ≥35 weeks. There are concerns that the encephalopathy exam that might be physiological normal but scored as abnormal (i.eg., suck and Moro reflex, posture) in babies of lower gestational age due to immaturity, there are concerns that TH in preterm patients <35 weeks may result in hypotension, increased oxygen consumption, and respiratory compromise as a result of reduced surfactant production.^41^ In this study we excluded RCTs with infants <34 weeks gestation. However, in the literature, overall complication rates from TH were similar among those born at 34–35 weeks’ GA compared to those born at >36 weeks’ GA, although hypoglycemia, rewarming before the completion of TH, mortality and white matter injury on MRI were higher in the earlier preterm group.^42^ Lower birth weight (LBW) was a much less frequently employed exclusion criterion, used in just 44% of included studies. Threshold values ranged from <2500 g to <1800 g. There are concerns for TH in LBW patients as hypothermia has been associated with an increased risk of IVH and an increased risk of death in the VLBW (<1500 g).^43^ Reassuringly, recent evidence suggests that BW for GA does not appear to influence complication rates or outcomes in TH (med.stanford.edu/pediatrics/research/trials/_jcr_content/main/panel_builder_0/panel_0/clinicaltrials.html?ctid=NCT01793129&conditionId=&serviceLineId=&condition=&alpha=p).^44^ RCTs of selective head cooling, employing an exclusion threshold BW of <1800 g, have also demonstrated a significant positive correlation between lower birth weight and better outcomes.^45^ The ongoing Preemie Hypothermia in NE^46^ is an NICHD trial and is a randomized trial of targeted temperature management with whole body hypothermia for moderate and severe hypoxic-ischemic encephalopathy in premature infants 33–35 weeks gestational age (NCT01793129) and will provide more information on TH in this group of infants.

There was significant variation in control group mortality between studies, varying between 2 and 67% between all studies, and between 27 and 39% even between the largest trials of TH with >100 participants included. Furthermore, since the broad introduction of TH as routine treatment in high-resource settings, there has been an extension of this intervention to patients that were not represented in the trials of TH, such as patients with mild NE/HIE/PA.^47^ There is increasing regarding the possibility of harm from treating with TH outside of the originally intended cohorts, such as increased apoptosis in animal experiments of cooling in uninjured brains, bed capacity in NICUs, and separation of mother and baby.^17^ Further, when the eligibility criteria of RCTs does not agree with the subsequent real-world treatment group, factors outside of the intervention itself may influence the measured effect of the intervention, called heterogeneity of treatment effect (HTE). This limits the generalizability of results.^48^ Therefore, it is even more difficult to generalize results from RCTs when the population differs across different studies, even when differences in eligibility criteria are subtle and the intervention is the same. As the eligibility for the intervention, TH, and more generally for the diagnosis of NE/HIE/PA is based on the eligibility criteria from RCTs, this issue is particularly significant for patients with NE/HIE/PA.

Comparison of the eligibility criteria employed in the large trials of TH demonstrated consistency across many criteria. However, control group mortality still varied significantly. As the study by Walsh et al. shows,^19^ small variations in application of eligibility criteria have major consequences and may partially explain this significant variation in control group mortality. There are too many variables and too wide variation in control group mortality for the entire cohort to make any definitive conclusions. It was beyond the scope of this study but a measure of the association between individual eligibility criteria and the control group mortality would indicate the strength of the association between the criterion and adverse outcome and, therefore, a measure for its justification for inclusion as an eligibility criterion in future trials. Recently a core outcome dataset has been published to harmonize future clinical trials for NE and facilitate consensus on reporting and data synthesis such as systematic reviews and meta-analysis. This COHESION core outcome set was developed with a broad international team involving parents and health care workers from high-, low-, and middle-income countries and included seven outcomes: survival; brain injury on imaging; neurological status at discharge; cerebral palsy; general cognitive ability; quality of life of the child, and adverse events related to treatment. Further refinement of these outcomes including standardized tools for measuring them are planned.

We chose to limit the study to RCTs as these studies have the most rigorously defined participant eligibility criteria and as the participant criteria from the most prominent studies are frequently used as case definition criteria for patients with NE/HIE/PA. We also chose this restriction understand the difficulties with heterogeneity in participant criteria specifically in relation to RCTs. Despite this, we recognize that limiting the study to RCTs may omit valuable information from observational or interventional studies of other design types.

## Conclusion

This systematic review was the first stage in developing a protocol for the definition. We have demonstrated significant variation in the terminology used to describe the condition of NE/HIE/PA and the case definition criteria in clinical trials. Consistent use of terminology, a common understanding of the condition, and how to diagnose it are urgent issues for future research.^7,20^ The variety in definitions used for the condition affects the comparability of trial results, advancements in knowledge in the field, development of neuroprotective strategies and clinical practice. Maintaining a broad but meaningful definition of the condition would provide an inclusive approach while permitting subclassifications within the condition, such as mild NE/HIE/PA, inflammation-sensitized NE/HIE, sentinel event HIE, and permitting comparisons and benchmarking across settings such as low-middle and high resource settings. Case definition criteria for clinical trials are always likely to be more restrictive than those for observational studies, such as prognostic studies, and may require different case definition criteria. Developing consistency across these areas, as far as possible, would allow improved interpretation of interventions on long-term prognosis and greater generalizability of trial results. Major efforts to date, including those by the ACOG/AAP Task Force, still need to resolve these issues and it may require a different approach to ensure further progress is made. Delphi methodology’s consensus-building approach may engage broader stakeholder groups and develop greater consensus.^49^

## Supplementary information

Supplmentary Table S1

PRISMA checklist definitions paper
